# Probing polydopamine adhesion to protein and polymer films: microscopic and spectroscopic evaluation

**DOI:** 10.1007/s10853-017-1806-y

**Published:** 2017-11-15

**Authors:** David Mallinson, Alexander B. Mullen, Dimitrios A. Lamprou

**Affiliations:** 1 Strathclyde Institute of Pharmacy and Biomedical Sciences, University of Strathclyde, Cathedral Street, Glasgow, G4 0RE UK; 2 Medway School of Pharmacy, University of Kent, Medway Campus, Anson Building, Central Avenue, Chatham Maritime, Chatham, Kent ME4 4TB UK

## Abstract

Polydopamine has been found to be a biocompatible polymer capable of supporting cell growth and attachment, and to have antibacterial and antifouling properties. Together with its ease of manufacture and application, it ought to make an ideal biomaterial and function well as a coating for implants. In this paper, atomic force microscope was used to measure the adhesive forces between polymer-, protein- or polydopamine-coated surfaces and a silicon nitride or polydopamine-functionalised probes. Surfaces were further characterised by contact angle goniometry, and solutions by circular dichroism. Polydopamine was further characterised with infrared spectroscopy and Raman spectroscopy. It was found that polydopamine functionalisation of the atomic force microscope probe significantly reduced adhesion to all tested surfaces. For example, adhesion to mica fell from 0.27 ± 0.7 to 0.05 ± 0.01 nN nm^−1^. The results suggest that polydopamine coatings are suitable to be used for a variety of biomedical applications.

**Electronic supplementary material:**

The online version of this article (10.1007/s10853-017-1806-y) contains supplementary material, which is available to authorized users.

## Introduction

The ideal coating for a biomedical device should be non-toxic, non-inflammatory, resistant to bacterial colonisation and support growth of host cells to improve integration [1]. The use of a coating allows the device interface to have favourable properties with the biological environment, even if the coating material is not the most appropriate for the core, exemplified by coated stainless steel cardiovascular stents which have appropriate biomechanical properties of the metal core. Additionally, a coating is particularly useful if it can be applied to the surface easily and uniformly. The mussel adhesive-inspired polydopamine (PD) appears to be an ideal biomaterial coating since it fulfils all these criteria. It exhibits low cytotoxicity [2], antibacterial properties [3, 4], promotes cell growth [5–8], resists corrosion [9], can aid antibiotic release [10, 11], is simple to produce with control over film thickness [12] and can be attached to a variety of surfaces including silanes [13], metals [9, 14], polymers [7, 15–17] dentine [18] and even hair [19].

Zhong et al. [20] found that while PD functionalisation of TiO_2_ nanotubes improved attachment, proliferation and nitric oxide release of endothelial cells, the same functionalisation reduced attachment and proliferation of smooth muscle cells. Despite promotion of cell attachment, modification of PD with polymer carpets, such as poly (3-sulphopropyl methacrylate) (PSPMA), can also be used to confer antifouling properties [8]. The ability to support cell attachment and growth is likely, at least partly, to be a result of its good hydrophilicity. For example, Hafner et al. [8] showed that PD nanosheets had a water contact angle (*θ*_A_) of 35°. Moreover, PSPMA surfaces are negatively charged; negatively charged fibroblasts resisted cell attachment. Other research has similarly been shown to lower the water contact angles of poly(tetrafluoroethylene) (PTFE) from 108.5° to 58.7° [21, 22] or 46.5° [23] poly(dimethylsiloxane) (PDMS) from 104.4° to 65.5° [21, 22] and titanium from 68.6° to 44.3° [21, 24]. This hydrophilicity-conferring property and propensity for modification make PD an ideal biomaterial and as such has been considered for tissue engineering applications and implants [9]. PD’s antibacterial properties appear to be due to the fact that monomeric dopamine can be cytotoxic, but polymeric PD is devoid of such activity. Hong et al. [2] found that some of the dopamine in the PD remains unpolymerized, but trapped within the PD matrix and can be released over time to provide an antibacterial effect.

The generation of PD is typically through the simple process of self-polymerisation/auto-oxidation of dopamine in a slightly basic environment [25]. The final product can be applied as a coating on a wide range of surfaces including metals, polymers and glasses [21]. The substrate can be removed after functionalisation if desired, with hydrofluoric acid or ammonium fluoride [8, 26], for example, to produce a nanosheet [8]. Despite the ease of its production, the structure of PD remains elusive, though Hong et al. [2] report that it is the result of both physical self-assembly and covalent oxidative polymerisation of dopamine and 5,6-dihydroxyindole (the oxidative product of dopamine). The self-polymerisation method creates small spherical particles of PD with a range of sizes (100–500 nm) which aggregate on surfaces into larger particles (0.5–5 µm) to provide thicker films, with higher self-polymerisation temperatures providing smaller particles and thinner films [27, 28]. The rate of polymerisation/oxidation has also found to be dependent on the dopamine concentration, oxygen concentration and pH [27].

Despite previous experiments with PD-coated particles and surfaces, the adhesive forces between PD and various surfaces have yet to be quantified. This research investigates the adhesion to protein-, polymer- and PD-coated surfaces via atomic force microscopy. Serum albumin, mucin and trypsin were used as model proteins.

Mucins are heavily glycosylated proteins forming mucous layers found throughout the digestive and pulmonary systems forming a protective physical barrier for mucosal surfaces. Though experiments have been performed with PD and mucin (MUC1) antibodies [29], to the authors’ knowledge no other PD mucin adhesion experiments have been performed. However, it was expected that adhesion to mucin would be low since a PEG-PD coating was found to reduce mucin adsorption even though the effect of PD alone was not significant; [18] and low mucin–mucin adhesive forces were seen by Berry et al. [30]. This agrees with Dague et al. [31], who observed that *Lactococcus lactis* adhesion decreased after mucin adsorption. PD has been considered for use in oral formulations which benefit from the effect of pH on its release of encapsulated drugs [32], so it is of interest to investigate its interactions with mucin.

Albumin is the principal blood plasma protein and plays a role in maintenance of osmotic pressure and transport of critical biomolecules, such as hormones [33]. Albumin is therefore relevant to understand interactions with vascular stent devices. Zelasko-Leon et al. [29] have previously immobilised MUC1 antibodies and albumin to gold nanorods via a PD coating which could suggest that BSA-PD adhesion, or at least adsorption, does occur. Human serum albumin has been observed to reduce the size of PD aggregates formed on a surface [34].

Trypsin is a proteolytic enzyme found in vertebrates’ digestive systems so is relevant for devices such as diagnostic intestinal imaging cameras and orally administered drug formulations. Koutsopoulos et al. [35] investigated trypsin adsorption to polystyrene (PS) and to silica and found that its affinity for hydrophobic PS was greater than for hydrophilic silica. Since PD has exhibited hydrophilic tendencies, it may again be expected that PD trypsin adhesion may be low. Despite this, it is not clear what the difference will be, since the unfunctionalised silicon nitride probe used as the control is also hydrophilic with a water contact angle of 32 ± 12° [36]. Therefore, it is important to understand the interactions of PD with synthetic materials, such as polymers used in biomedical applications.

Poly(ε-caprolactone) (PCL), poly (L-lactic acid) (PLLA) and poly (2-hydroxyethylmethacrylate) (PHEMA) were used as test polymers because they have applications as biomaterials due to their corrosion resistance, cytocompatibility and degradability that allows drug release [37, 38]. PCL fibres have been coated with PD and investigated for use as bone tissue scaffolds [39], and 3D-printed scaffolds of PCL have been coated with PD resulting in an improvement in cell density versus control [17]. PLLA can be used for tendon repair [40], as a coating for stents [41] or made into scaffolds and coated with PD which improved adhesion and cell proliferation [7, 42]. PHEMA has uses as a tissue engineering scaffold, sometimes as a hydrogel [38], and for the immobilisation of proteins [43].

In this investigation, the adhesive forces between PD-, protein- or polymer-coated surfaces and bare silicon nitride or polydopamine-functionalised AFM probes are measured. Surfaces are also characterised with AFM for roughness (*R*_a_) and morphology, with contact angle goniometry (CAG) for hydrophobicity and surface energy and with RAMAN and FTIR for spectroscopic evaluation.

## Materials and methods

### Materials

Dopamine hydrochloride (H8502) was purchased from Sigma (Germany), and Tris (hydroxymethyl) aminomethane (Tris; ≥ 99.8%) from Sigma Aldrich (USA). Porcine gastric mucin type II (PGM; Sigma M2378), bovine serum albumin (BSA; Sigma A7906), trypsin (Sigma T4549), diiodomethane (DIM; 99%) and phosphate-buffered saline (PBS; pH 7.4) tablets were purchased from Sigma (USA). Ethylene glycol (EG; 99.8%) was purchased from Sigma Aldrich (UK), and dichloromethane (DCM), N-dimethylformamide (DMF) and 3-aminopropyltriethoxysilane (APTES; 99%) were all purchased from Sigma Aldrich (China). PLLA (M_W_ ~ 55000 g mol^−1^) was purchased from Aldrich (Germany), and PCL (M_W_ 14000 g mol^−1^) and PHEMA (M_V_ 20000 g mol^−1^) were purchased from Aldrich (USA).

### Preparation of substrates and AFM cantilevers

**Figure 1:**
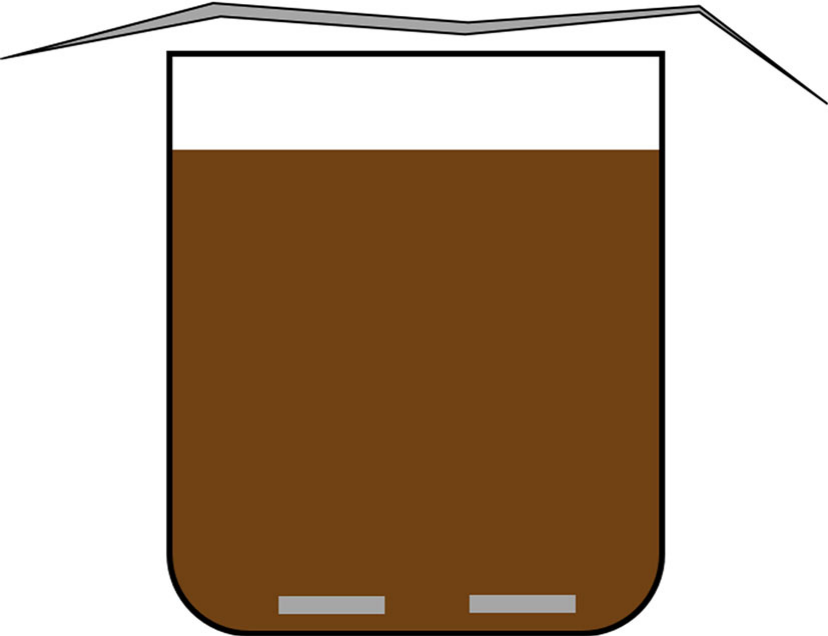
Production of PD-coated SW. Squares of SW were left in solution overnight and loosely covered with foil to keep dust out but allow oxygen in

PD was grafted onto surfaces by immersion in a basic (pH 8.5) solution of 10 mM Tris and 2 g L^−1^ (10.5 mM) dopamine hydrochloride in DW overnight (~ 18 h) as per previous groups (Fig. 1) [25, 44–46]. AFM probes were APTES-functionalised in a manner equivalent to Hong et al. [47] and Lyubchenko et al. [48], to act as a primer to further functionalisation; the probes were left in APTES vapour for at least 30 min. Though PD is a good coating material for surfaces, APTES may ensure that it stays attached throughout the experiment. The probes were removed from the vapour and then washed with DW. Thereafter, the PD functionalisation was as above.

Drop casting was used to coat silicon wafer (SW) with polymers other than PD. Solutions were made with a polymer concentration of 2% w/v. PLLA and PCL were dissolved in DCM, while PHEMA was dissolved in DMF. Polymer solution was added to squares of SW (10 × 10 mm^2^). Solvent was allowed to evaporate in a fume cupboard until dry and samples were stored in a desiccator until measurement. Drop casting was also used to coat SW with protein. BSA and PGM were each dissolved in PBS to 3.0 mg mL^−1^. After vortexing, a maximal volume (as above; up to 300 µL cm^−2^) was placed on a square of SW (10 mm × 10 mm). This was allowed to dry in a fume cupboard overnight. A large range of concentrations have been used previously in our laboratory in order to produce protein films, and this combination of concentration and volume was found to be sufficient to leave a visible deposit on the surfaces.

### Contact angle and surface energy measurements

To probe liquid–surface interactions, contact angles (*θ* at 20 °C) of small drops (typically 2–10 µL; 2–3 drops per substrate and 2–3 substrates per sample) of deionised water (DW; Millipore, 18.2 MΩ cm; surface tension *γ*_L_ = 73.5 mN m^−1^ at 15 °C [49]), DIM (> 99%; *γ*_L_ = 50.8 mN m^−1^ at 20 °C [50]) and EG (> 99%; *γ*_L_ = 47.7 mN m^−1^ at 20 °C [50]) placed on horizontal substrates were measured using a Krüss DSA30B contact angle goniometer (CAG) with Krüss ADVANCE drop analysis software. Static contact angles (*θ*; ± 0.1°) were obtained for both left and right contact angles 5 s after placement of the drop. Surface energies of substrates (*γ*_s_) were calculated from the contact angles and the interfacial energies of the three probe liquids using the Good and Oss [51] three-liquid formula (Eqs. 1, 2 and 3), using an in-house Visual Basic program as per Lamprou et al. [52]. Contact angle measurements were performed after AFM or on alternate surfaces in order to ensure that the drops dip did not affect the surface morphology. Because mica’s water contact angle proved too low to accurately measure on our apparatus, a value of 10° was chosen. The value has previously been shown to be < 10° [53].

1 $$ \gamma_{\text{S}} = \gamma_{\text{S}}^{\text{LW}} + \gamma_{\text{S}}^{\text{AB}} = \gamma_{\text{S}}^{\text{LW}} + 2\left( {\gamma_{\text{S}}^{ + } \gamma_{\text{S}}^{ - } } \right)^{1/2} , $$

2 $$ \gamma_{\text{L}} = \gamma_{\text{L}}^{\text{LW}} + \gamma_{\text{L}}^{\text{AB}} = \gamma_{\text{L}}^{\text{LW}} + 2\left( {\gamma_{\text{L}}^{ + } \gamma_{\text{L}}^{ - } } \right)^{1/2} , $$

3 $$ \gamma_{\text{L}} (1 + \cos \theta ) = 2\left[ {\left( {\gamma_{\text{S}}^{\text{LW}} \gamma_{\text{L}}^{\text{LW}} } \right)^{1/2} + \left( {\gamma_{\text{S}}^{ + } \gamma_{\text{L}}^{ - } } \right)^{1/2} + \left( {\gamma_{\text{S}}^{ - } \gamma_{\text{L}}^{ + } } \right)^{1/2} } \right], $$

where superscripts denote components of surface energy, LW Lifshitz–van der Waals, AB acid–base, *γ* ^+^ Lewis acid and *γ* ^−^ Lewis base.

### Atomic force microscopy

A Bruker Multimode 8 atomic force microscope with Nanoscope V controller was used for the AFM measurements. Measurements were made in air under ambient conditions. Spring constants and resonant frequencies for the cantilevers were determined with the thermal noise method at the start of each experiment. Force–distance plots were obtained at random locations on the sample using a V-shaped ScanAsyst air probe (nominal spring constant 0.4 N m^−1^; nominal resonant frequency 70 kHz; nominal length 600 nm) in force–volume which performs ramps in a grid. Force curves were selected at random from a 32 × 32 array. Adhesion forces (F_a_; n = 80–90) were extracted from the force curves (example given in Supplementary Information 1) using a on house prepare Python script, previously used in Mallinson et al. [54]. Forces were then normalised for tip radius by dividing the force by tip radius proportional to Sugawara et al.’s [55] correction shown in Eq. 4.

4 $$ A = 4\pi RT, $$

where *A* is corrected adhesion, *R* tip radius and *T* medium surface tension.

The tip radius (*R*) was determined by scanning (scan size 5 µm, scan rate 1 Hz) an etched silicon surface (PA01, MikroMasch, San Jose, CA, USA) in peak force-quantitative nanomechanical mapping (PF-QNM) mode followed by using the tip qualification function within Nanoscope Analysis. Surface morphology was imaged in PF-QNM mode. The surface roughness (*R*_a_) of each substrate was determined by using Nanoscope Analysis’ algorithm to analyse several scans of the surface from different locations.

### Circular dichroism

Circular dichroism (CD) was used to probe the secondary structure of the proteins and attempt to identify any secondary structure in the PD. Spectra were read in the wavelength range of 190–280 nm with a Chirascan Plus spectrophotometer (Applied Photophysics). A step size of 1 nm, bandwidth of 1 nm and reading time of 1 s per point were chosen. The samples were placed in a quartz cuvette (Hellma) with a path length of 0.1 mm. DW (Millipore) was used as the background. Whole PD, the product of the above process, and the same solution passed through a 0.2-µm syringe filter such that no particles were visible were used as samples. Three spectra were recorded for each sample, averaged, smoothed and the background subtracted. Data were processed with Chirascan Viewer and Microsoft Excel.

### Spectroscopic analysis

Fourier-transform infrared spectroscopy (FTIR) spectra were taken for dopamine hydrochloride and PD. PD, as prepared above, was centrifuged at 2000 rpm for 5 min, and then supernatant was removed. The pellet was spread on a glass slide and allowed to dry before measurements. Spectra were recorded with a Bruker Tensor II FTIR spectrometer in the wavenumber range 400–4000 cm^−1^. Each sample spectrum was made of 16 scans.

Raman spectra were taken for dopamine hydrochloride and PD, prepared as for FTIR, using a Horiba Scientific XPloRA Plus Raman microscope calibrated with SW. Measurements were taken with a green (532 nm) laser in the wavenumber range of 50–3600 cm^−1^ with a grating of 1800 gratings mm^−1^, a 100 µm slit and a × 10 objective lens. Spectra were recorded at three locations per sample and the backgrounds removed.

### Statistics

Data were processed with Microsoft Excel 2016 and Minitab 17. Pairwise differences in adhesion results were analysed with a series of 2-sample *t* test with a significance level of 0.05.

## Results and discussion

### Surface characterisation

After PD functionalisation, surfaces were visibly different, displaying a black-sepia tint that remained even after washing with DW. The PD layer could be expected to be approximately 50 nm thick which is the approximate thickness attained after immersion for 18 h seemingly regardless of substrate according to Liu et al. [12] and Lynge et al. [56]. The water contact angle (Table 1) for PD-coated SW was low (16.8 ± 4.0°), indicating high hydrophilicity and showed good similarity with Perikamana et al.’s [21] values for PD-coated PLLA-hemp composites (16.9 ± 1.6°). This could be due to the coating being powder like. However, an even more extreme example was seen by Ku et al. [6] who observed a water contact angle of 0°, i.e. complete wetting, for PD-functionalised poly (lactic-co-glycolic acid) (PLGA) nanofibres.

**Table 1:** Surface characterisation: contact angles (*n* ≥ 6) and surface energies by CAG, and roughness (*R*_a_) by AFM

	Contact Angle (*θ*, °)			Surface energy (mJ m^−2^)				Roughness (nm)
Surface	DW	EG	DIM	*γ*_s_^+^	*γ*_s_^−^	*γ*_s_^LW^	*γ*_s_	*R*_a_
PHEMA	52.7 ± 1.9	64.5 ± 2.8	32.3 ± 1.2	2.53	50.21	43.24	65.77	4.3 ± 4.1
PLLA	94.8 ± 7.3	70.5 ± 1.9	56.4 ± 2.1	0	2.12	30.65	30.72	41.7 ± 14.8
PCL	93.1 ± 2.5	65.3 ± 2.5	32.9 ± 1.2	0.18	1.41	42.98	43.98	39.4 ± 4.7
PD	16.8 ± 4.0	20.1 ± 1.9	54.6 ± 4.6	0.51	67.17	31.68	43.33	9.8 ± 0.5
Mica	< 10	33.8 ± 2.5	37.2 ± 1.8	0.16	77.12	47.95	47.91	0.4 ± 0.2

**Figure 2:**
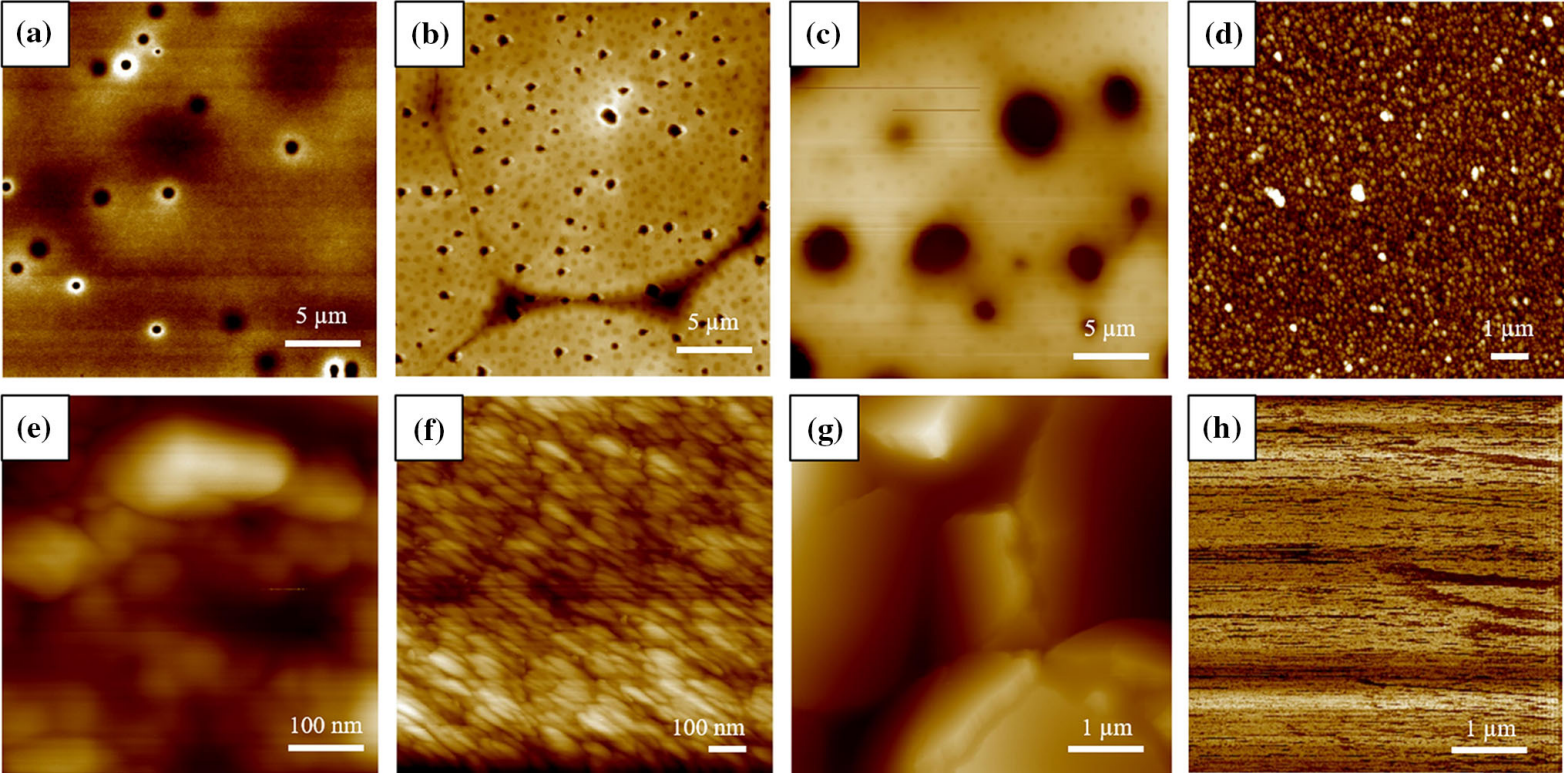
Morphology of surfaces by AFM: **a** PHEMA; **b** PCL; **c** PLLA; **d** PD; **e** PGM; **f** BSA; **g** trypsin; **h** mica

The water contact angle for PCL was higher than recorded by other groups—93.1 ± 2.5° compared to 74° [57] or 82.7 ± 3.2° [58]—as was the water contact angle for PHEMA—52.7 ± 1.9° compared to ~ 20° [59]—though the PLLA water contact angle was in agreement with other recorded values—94.8 ± 7.3° compared with 98.0 ± 2.3° [60]. The difference may be due to topographical differences since the dimpled textures (Fig. 2a–c) can increase hydrophobicity though this does not appear to have had the same effect upon PLLA’s water contact angle.

### Morphology

The images in Fig. 2 are shown at scales that best display the features observed. The polymer films (Fig. 2a–c) show flat but featured surfaces. Dimples can be seen on the films which are likely from condensed water droplets acting as a template for the polymer as it was cast by the evaporating solvent. This is similar to the process for breath figure array formation—where water droplets condensing as the solvent evaporates act as a template for the polymer as it is cast—but here the pores lack order, likely due to the dry environment in which they were cast. With a similar appearance to the PD previously observed via AFM [5], the image of deposited PD (Fig. 2d) shows a majority of small particles (100–250 nm) with some larger aggregates (350–550 nm) which fall in the range of sizes observed by others [5, 27, 28]. This sample was found to be smoother (*R*_a_ 9.8 ± 0.5 nm) than reported values from Bi et al. [23]. (*R*_a_ 35.8 ± 2.1 nm) though the substrate of SW in our studies was smoother (*R*_a_ 0.15 ± 0.04 nm [61]) than the previously reported PTFE substrate (24.2 ± 1.3 nm) [23]. The dried protein films (Fig. 2e–g) show textured surfaces with PGM (e) and BSA (f) having rounder features than the relatively ‘mountainous’ trypsin (g). Mica (Fig. 2h), on the other hand, is very flat–the z-axis was on the order of nanometres.

### Adhesion

**Figure 3:**
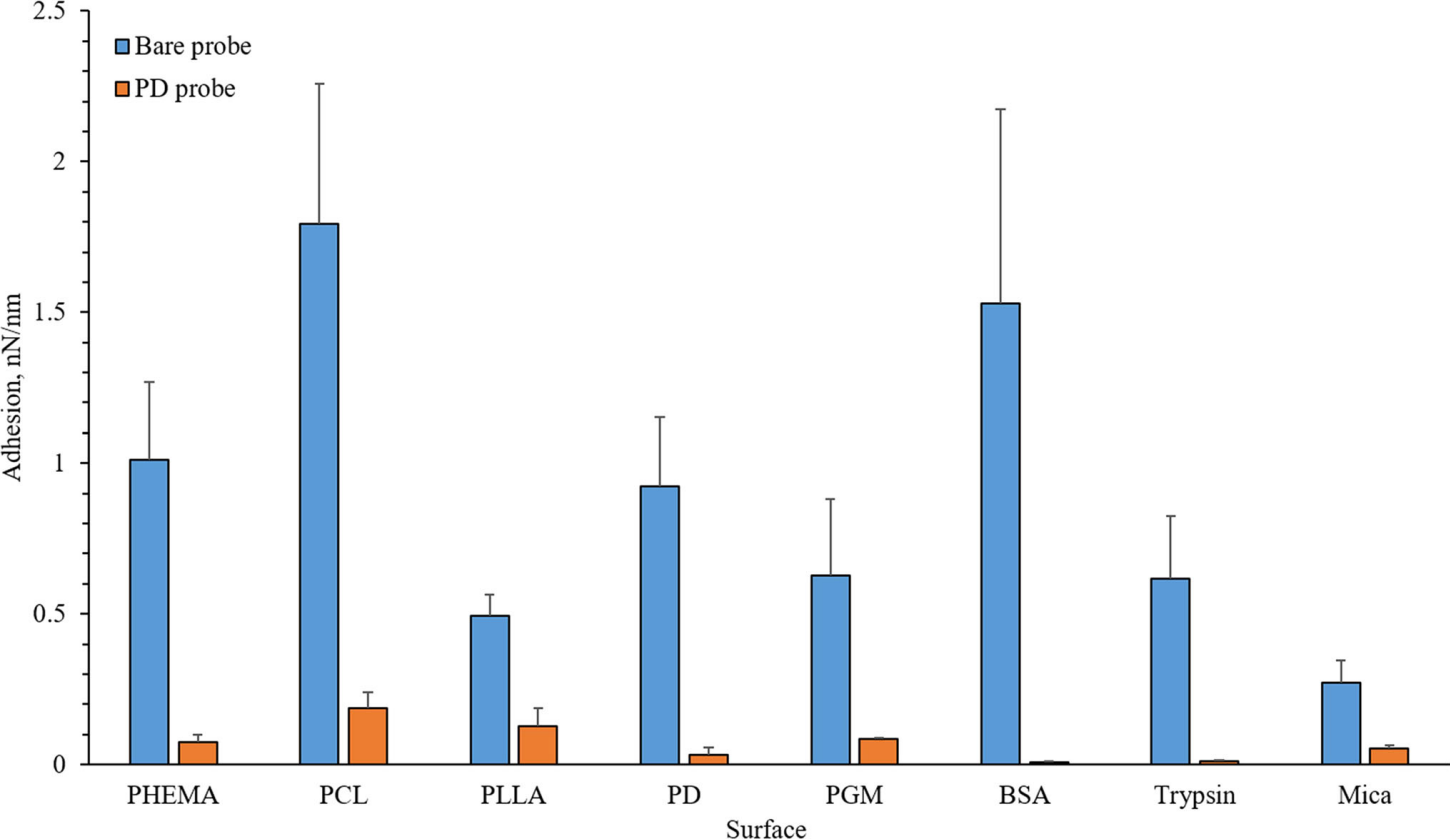
Mean adhesion (± SD) to different surfaces with bare or PD-functionalised AFM probes adjusted for tip radius. All pairs are significantly different (*P* < 0.001)

When the forces are adjusted to the force tip radius, all adhesive forces recorded with a PD-functionalised probe are significantly (*P* < 0.001) lower than with a bare, silicon nitride probe (Fig. 3). Since it has been found that PD supports cell growth [5], it might be expected that the proteins would experience more adhesion though the opposite appears to be true even when the unadjusted values are considered (Supplementary Information 2) except for mucin.

The low adhesion observed with the PD-functionalised probes appears contradictory to the ease that PD can functionalise surfaces, as enabled by the displayed catechol, imine and amine groups [32]. The apparent low adhesion to protein films suggests that it may be ideal as a biomaterial or biomaterial coating by minimising interactions with proteins. However, these experiments took place in a dried environment and a solvent (e.g. PBS or serum) might have an effect on the adhesion and thus on the force–distance plots that have been measured. Therefore, adhesion forces might change in wet environment.

### Circular dichroism

**Figure 4:**
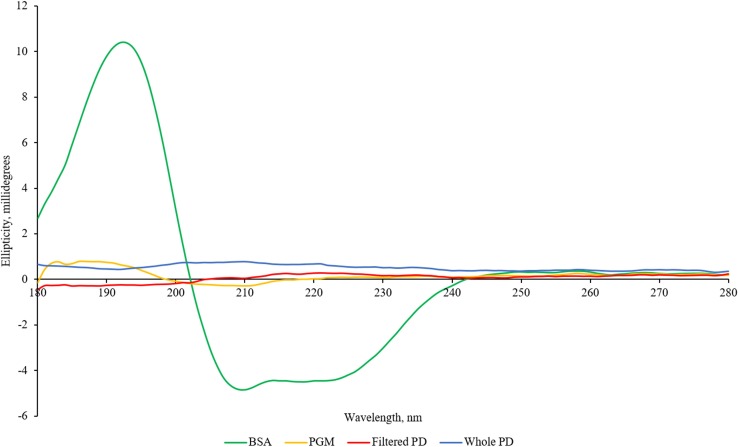
Circular dichroism spectra of PD, BSA and PGM

Due to the structure PD assumes when it self-polymerises [2] it is expected that it would be unlikely to display peaks that are synonymous with protein secondary structure noticeable by CD and indeed it does not since it is not a polypeptide. Although there do not appear to be any spectra of PD in the literature, Zelasko-Leon et al. [29] show a spectrum of PD-functionalised gold nanorods which exhibits no positive or negative maxima. The filtered PD product had a sepia colour, but had no visible particles. Neither this filtered PD product nor the unfiltered, whole PD product gave any discernible signal above the background (Fig. 4).

The crystal structure of BSA (Protein Data Bank (PDB) 4F5S) [62] shows an abundance of alpha helices. The negative and positive maxima at 208 nm and 193 nm, respectively, seen in Fig. 4 are in very good agreement with what is expected for alpha helices [63]. Alpha helices also show negative ellipticity at 222 nm which may be present in the spectrum. This suggests that the protein retained its expected structure when reconstituted.

Mucins tend to have very little beta secondary structure and even less alpha secondary structure and are instead mostly random coil [64]. This could be reflected by the very weak ellipticity that is barely above the background.

Trypsin contains some alpha helices and beta-pleated sheets (RCSB entries 5GXP [65] and 5JYI [66]). The spectrum reported in Supplementary Information 3 agrees with this by the positive maximum at 193 nm [63]. It also shows negative maxima at 197 nm which corresponds to random coil structures [67] as well as at 202 and 204 nm, but these do not appear to correspond to any obvious secondary structure. There is also a local negative minimum at 208 nm which may be due to alpha helices.

### Spectroscopic analysis

**Figure 5:**
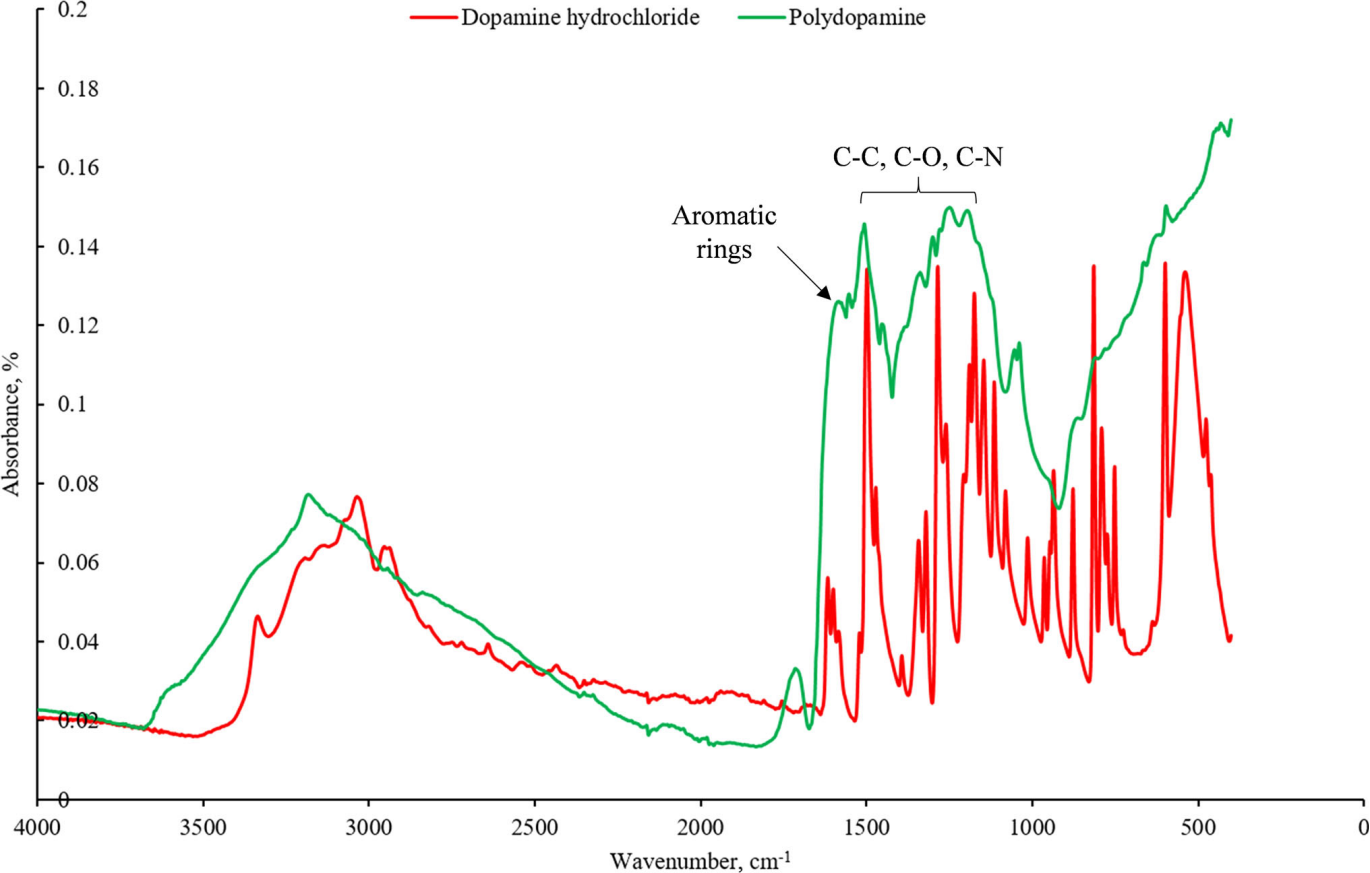
FTIR spectra of dopamine hydrochloride and the PD product

The FTIR spectrum (Fig. 5) for dopamine hydrochloride is consistent with spectra gathered by other groups [68]. A peak that is common for both dopamine hydrochloride and PD near 1500 cm^−1^ may be attributed to aromatic regions that are common in both structures. The spectrum for the PD product appears to resemble the spectrum that would be achieved by overlaying the spectra for dopamine hydrochloride and Tris base, and also shows similarity to spectra obtained by Iqbal et al. [4] for PD with the 1200–1500 cm^−1^ region being assigned to C–C, C–O and C–N, and the 3000 cm^−1^ region assigned to C, N, O and 3500 cm^−1^ to primary amine stretching which is supportive of PD. There is also similarity to Mei et al.’s [18] spectrum with a common peak near 1600 cm^−1^ indicative of aromatic rings. There is further similarity to spectra obtained Iqbal et al. [69] for PD-coated nanoparticles and good similarity to the spectrum obtained by Steeves et al. [5] for PD with nanoporous titanium.

**Figure 6:**
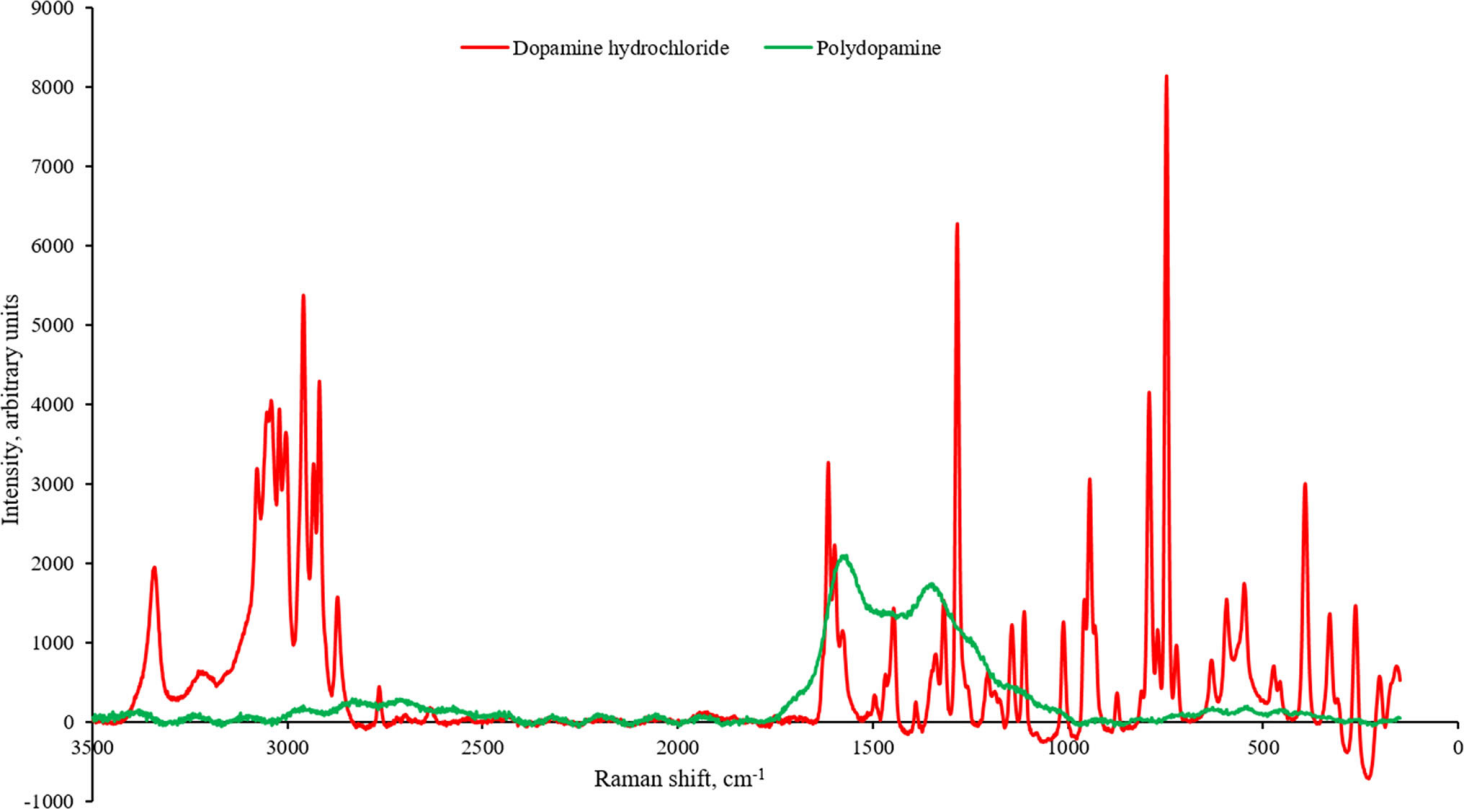
Raman spectra of dopamine hydrochloride and PD

Raman spectra were consistent between scans in different locations for the same samples (data not shown); however, the spectra (Fig. 6) for each material are different. The dopamine hydrochloride spectra show peaks typical of aliphatic chains (600–1300 cm^−1^), while the peaks (not shown) in the low wavelength range (10–200 cm^−1^) were suggestive of crystallinity. The dopamine hydrochloride spectrum matched that of the supplier [70]. The Raman spectra for PD show similarities to those obtained previously by other groups such as Ma et al. [71], Steeves et al. [5] for PD with nanoporous titanium, Lee et al. [9] for coated titanium and Qiu et al. [72] for MnCO_3_ with PD which show bands at 1350 and 1580 cm^−1^ which, as for dopamine hydrochloride, are suggestive of aliphatic and aromatic components [6, 71].

## Conclusion

PD has been considered as a biomaterial due to its ease of application and promotion of cell growth. The product was found to resemble the PD produced by previous groups. By atomic force microscopy, PD-functionalised probes to protein films and polymer films were significantly lower than with an unfunctionalised silicon nitride probe, suggesting that a PD could be a useful coating for reducing interaction with protein that may otherwise lead to fouling. This supports the trend towards using PD as a coating for biomedical devices.

## Electronic supplementary material

Below is the link to the electronic supplementary material.

Supplementary material 1 (DOCX 254 kb)
