# Supplementary material for: Probing polydopamine adhesion to protein and polymer films: microscopic and spectroscopic evaluation
Source: J Mater Sci. 2017 Nov 15;53(5):3198–209. doi: 10.1007/s10853-017-1806-y (PMC6956915; doi:10.1007/s10853-017-1806-y)

**Supplementary Information 1:** Example of an AFM force curve.

**Supplementary Information 2:** Mean adhesion (± SD) by AFM not adjusted for tip radius. All pairs are significantly different (p ≤ 0.001).

**Supplementary Information 3:** Circular dichroism spectrum of trypsin.


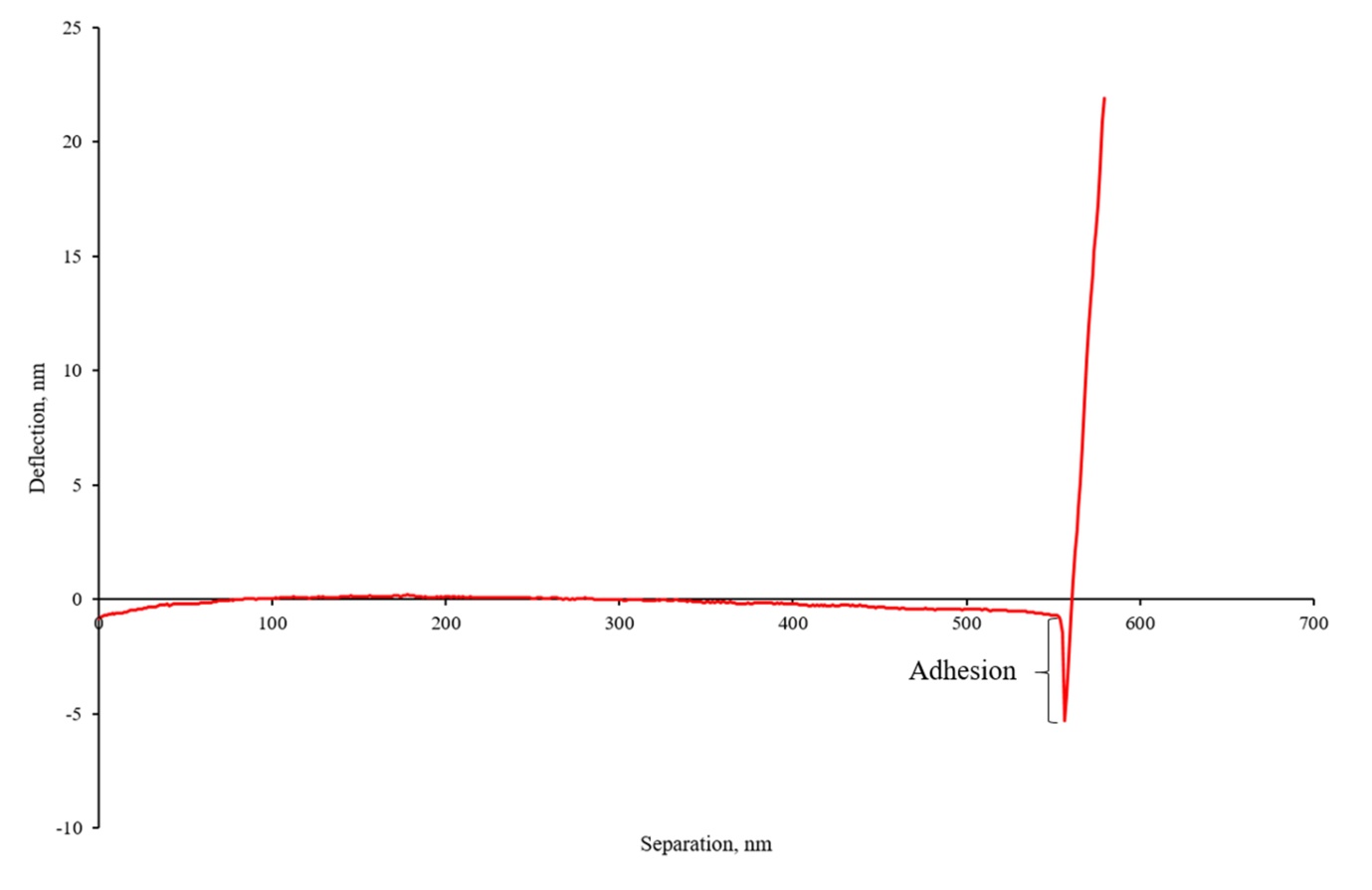


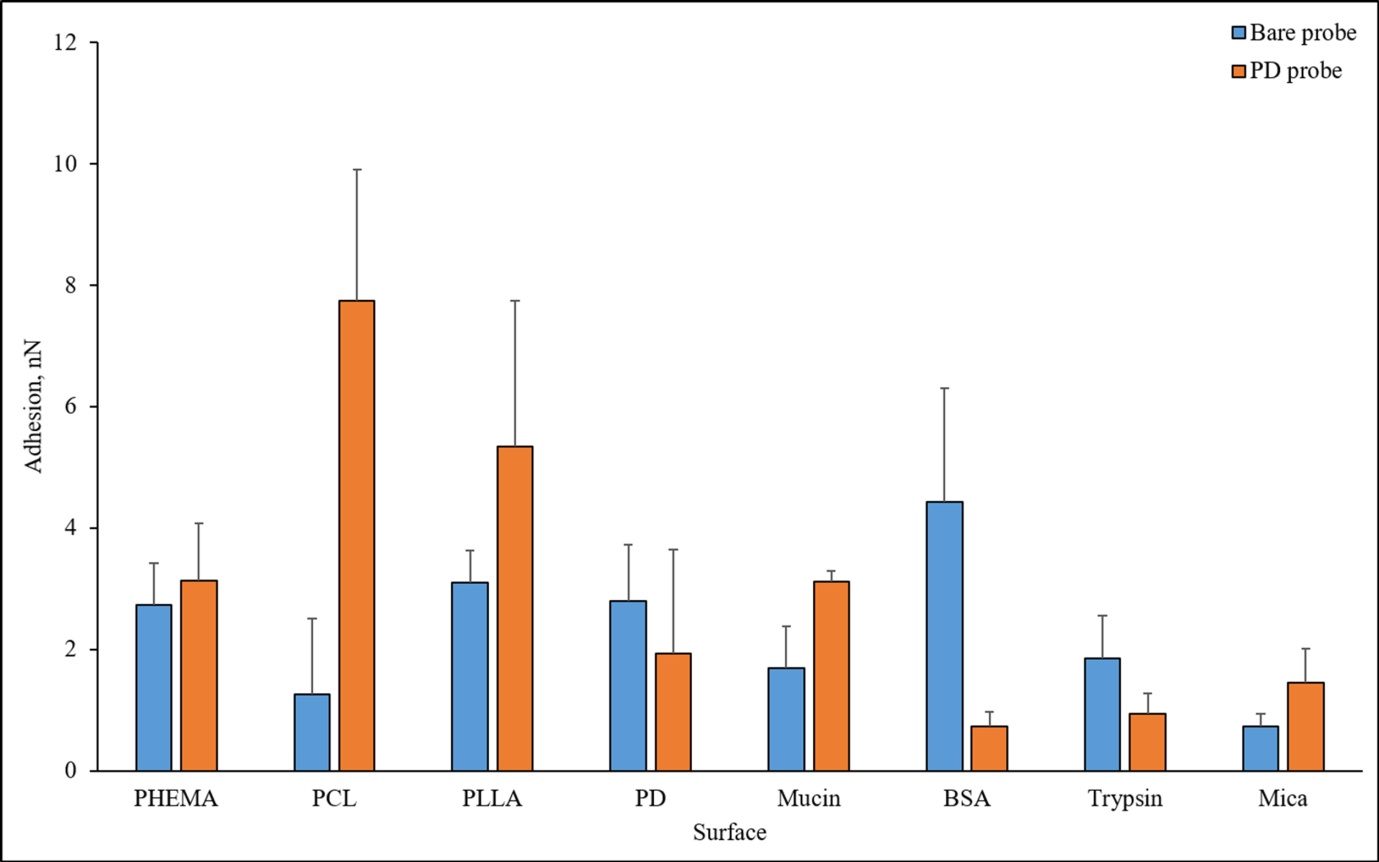


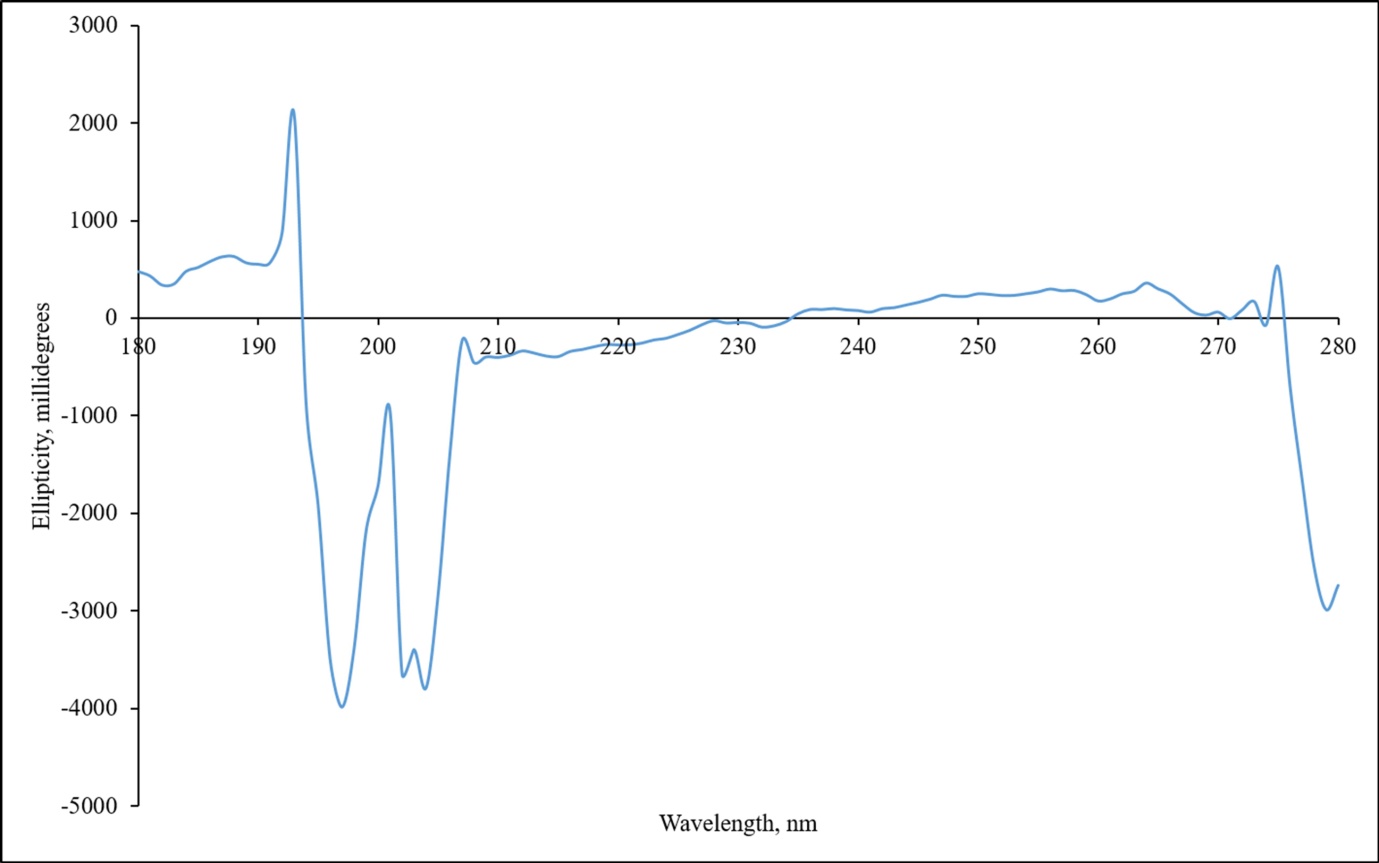

Supplement: Supplementary file 1 — Supplementary material 1 (DOCX 254 kb) [file 10853_2017_1806_MOESM1_ESM.docx]
